# Preclinical training of future ocular surgeons: a French opinion-based study

**DOI:** 10.1186/s12909-024-05124-8

**Published:** 2024-02-09

**Authors:** Nicolas Kitic, Jean-Louis Bourges

**Affiliations:** 1 Rothschild Foundation Hospital, Paris, France; 2 Sorbonne Université, Paris, France; 3 INSERM, UMRS1138, Team 17, From Physiopathology of Ocular Diseases to Clinical Development, Centre de Recherche des Cordeliers, Université Paris Cité, Paris, France; 4 Department of Ophthalmology, Cochin Hospital, AP-HP, Paris, France

**Keywords:** Surgical training, Simulation, Cataract surgery training, Ophthalmology residency

## Abstract

**Purpose:**

To assess ophthalmology residents satisfaction regarding surgical training during residency in France.

**Methods:**

A questionnaire consisting of 28 questions was designed and sent to residents of ophthalmology across the 27 French regions.

**Results:**

A total of 30.3% ophthalmology residents in France completed our questionnaire. All French regions participated. They rated 5,27 ± 2.42/10 the global surgical training during residency. They had performed at least one step of any type of ocular surgery for 93.4% of them, while 80.7% had completed at least one full cataract surgery, by the beginning of their second year of residency on average (Paris: 2.59 ± 1.36 semesters; regions: 4.05 ± 1.96 semesters, *p* < 0.0001). Only 48.9% identified a surgical mentor during their residency, but 82.2% did not clearly identify surgical goals & objectives during their training. Simulation was available for any type of ocular surgery to all residents in the Paris (Île-de-France) region and to 78.1% in other regions (*p* < 0.005). Residents who accessed drylabs and wetlabs gave a satisfaction score of 7.31 ± 1.89/10 and 6.39 ± 2.15/10 to it respectively. Simulation was a mandatory part of the curriculum for 35.2% of the resident. They commented on on reduced access to subspecialized surgery. They were willing for more access to simulation and surgery on real patients, as well for closer mentoring and clearly defined surgical goals within the curriculum.

**Conclusion:**

Ophthalmology residents seemed globally satisfied with their surgical training nationwide, although we observed disparities across region. They largely acknowledged a lack of standard procedures nationwide. They acclaimed simulation during the initial phase of residency, progressively switching towards surgery on real patients. An “operating license” during residency could be a viable way for the resident to demonstrate that they have acquired enough surgical abilities to perform surgery on real patients.

**Supplementary Information:**

The online version contains supplementary material available at 10.1186/s12909-024-05124-8.

## Introduction

Ophthalmology learning concomitantly associates theorical workout, clinical and surgical training. Not only cognitive loads participate in the know-how of specialty but also technical dexterity and mechanical sense, which is supposed to develop all along the initial training [1]. 

Medical training relies primarily on knowledge made of memory recalling, followed by clinical application, which generates procedural memory. Both are thereafter applied on patients [2]. In that, surgical skills extend far beyond intellectual knowledge.

Ocular surgery is particularly demanding because each procedure step shapes ineluctably the next one and can never be processed twice.

To ensure learning success for in-training surgeons, some supplemental background should be acquired as compared to exclusively medical specialties. For instance, students should master every technical detail and step of ocular procedures, practice with ease using both hands and feet, anticipate common reactions of biological tissues, accustom themselves to complex operating devices, and finally manipulate intraocular prosthesis and biomaterials.

Furthermore, ophthalmologists operate under operating microscopes as well as complex visualization systems, including augmented reality.

Finally, they should master surgical non-technical skills. Before proceeding on patients in real-life, self-confidence is mandatory and implies a significant commitment ahead of training.

In such a context, simulation seems an interesting option. It is positively correlated with surgical dexterity of resident and junior surgeons in real-life ocular surgery [3–5]. 

Practically, two fields of surgical training are usually associated: dry- or wetlab simulation, and hands-on training. In most French cities, simulation has been set available throughout the last decade, using virtual reality simulators (e.g.: *EyeSi surgical*, *Haag-Streit*; Germany) or basic surgical kits (e.g.: *Kitaro kits*, *FCI Ophthalmics*; MA, USA). Teaching program have been designed for simulation and were incorporated within residents training programs. Concurrently, senior surgeons have supervised junior surgeons in their hands-on training, at least while performing surgery on real patients. The way to emancipate future ocular surgeons should ideally lead them to progress from simulating surgery to hands-on training, under educational master supervision. In the end, in-training surgeons should be evaluated for surgical dexterity, ideally in a reproducible and impartial fashion. Such attributes are not entirely fulfilled solely by senior surgeon’s opinion. The contribution of objective scoring provided by simulators could dramatically help. Still, it remains optional in many European Union countries, including France.

Worldwide, Ophthalmology residents seem generally satisfied by their surgical training [6–8]. Recently, residents of ophthalmology from Paris reported a good satisfaction level toward the surgical side of their training program. At most, some of them suggested to further improve access to simulation and hands-on labs. Some others claimed being ill-prepared to ocular surgery in emergency eye-care [9]. But in fact, little is known about the global opinion of residents in ophthalmology and how they would rate the surgical program they are enrolled in. Not much data are available to compare resident’s feedback across regions within a single country.

We elaborated a dedicated questionnaire to address the question. We gathered opinions about surgical training programs during residency. We allowed spontaneous suggestions from residents regarding any possible improvement in the surgical program. We sent the questionnaire to all students enrolled in a French residency program of ophthalmology and are presenting the answers so collected. The aim of the present report is to present the opinion of French residents in ophthalmology toward their own surgical training.

## Methods

### Ophthalmology residency training in France

Residency programs in France last 6 years, divided in 3 phases:

- *First phase (“phase socle”)*: 1 year dedicated to learning the basic clinical skills in Ophthalmology as well as first simulation training sessions; a surgical simulation exam is offered in some regions at the end of this first year;
- *Second phase (“phase d’approfondissement”)*: 3 years of in-depth learning in which the resident is actively involved in patient care, with night calls, increased responsabilities in the operating room in the presence of a tutor;
- *Third phase (“phase de consolidation”)*: 2 final years of learning consolidation, in which the resident has increasing autonomy within patient care, the last year being more similar to fellowship.

After residency, one or two years are required as a fellow in order to be an Ophthalmologist and pursue a career in private practice or within a hospital.

No objective list of surgical procedures or skills is required at the end of residency. Surgical simulation is mandatory in some regions, optional in others and not available in a few regions.

There are no national guidelines regarding simulation requirements during residency, although centers offering simulation generally have a passing score of 400 on the *EyeSI simulator*.

### Questionnaire inception

We sent a questionnaire to residents enrolled in a French ophthalmology residency program. Residents where exhaustively identified across the 27 French regions, based on the public list of French residents, annually released by the Official Journal of the French Republic (*Journal Officiel de la République Française*). We managed to contact residents by email through mailing lists, by telephone and through social media platforms. We generated an online questionnaire using the software Google Forms®. The residents were given the opportunity to take the survey by following the questionnaire’s URL. The respondents had to be enrolled in a French residency program. The seniority could range from first to last year of residency. The questionnaire form consisted in 27 successive questions. It was meant to propose simple/multiple-choice or open-ended questions. The first part inquired about the geographical location of the residency program (city) and the starting year (i.e. first year of the program).

Next parts successively asked to residents rating from 0 to 10 their own surgical training program, to attribute the ideal proportion to simulation or hands-on training during their training, and whether or not they would identify a personal mentor, a list of items (goals & objectives) to achieve before the end of the program and should comply to a formal program of simulation (e.g. simulation clerkship for drylabs and/or wetlabs). We questioned whether simulation was an obligation to the teaching program. We inquired about the type of simulated surgery (stitching, incisions, cataract extraction, keratoplasty, filtering or vitreoretinal surgery, etc.). They should rate from 0 to 10 both drylabs and wetlabs, and whether they could attend to it. We also gathered the delay to complete their very first surgical procedure on a real patient, cataract extraction and vitreoretinal surgery. In the following part, we asked to self-evaluate their surgical autonomy after 4 years of residency (before the third phase of residency) and after the 6-years residency (after having completed the third phase). In the last part, some free comments could be provided regarding surgical training and teaching. Wishes could be formed for further improvement.

This study was approved by the Ethics Committee of the French Society of Ophthalmology (IRB 00008855 Société Française d’Ophtalmologie IRB#1).

All methods were carried out in accordance with relevant guidelines and regulations.

All experimental protocols were approved by by the Ethics Committee of the French Society of Ophthalmology.

Informed consent was obtained from all subjects and/or their legal guardian(s).

### Group of residents

Residency programs were grouped according to French regions for analysis as follows:

- Paris (Paris city, Ile-de-France);
- North (Amiens, Angers, Besançon, Brest, Caen, Dijon, Lille, Nancy, Nantes, Poitiers, Reims, Rennes, Rouen, Strasbourg, Tours);
- South (Bordeaux, Clermont-Ferrand, Grenoble, Limoges, Lyon, Marseille, Montpellier-Nîmes, Nice, Saint-Etienne, Toulouse);
- Overseas (Antilles-Guyane).

The term « other regions » referred to the pooled data from North, South and Overseas regions.

### Statistical analysis

We described continuous variables by means and standard deviations, and we compared them with a Student’s t-test, after the data’s distribution was verified for normality with a Shapiro-Wilk test.

A one-way analysis of variance (ANOVA) test was used to compare continuous variables if their number was superior to 2 variables.

We proceeded with categorical variables by percentages and compared them with a Chi-square test when required. All tests were bilateral, and we considered a *p*-value < 0.05 as statistically significant. Statistical analyses were performed with the Statistical Analysis System® (SAS v9.4) and figures were built using Microsoft Excel® software.

## Results

We reached by email or social media a total of 1057 residents. Among them, 321 answered the questionnaire, accounting for a global responding rate of a third (30.3%). Residents registered in a specific transversal program (subspecialty training within residency in France, available in Oculoplastics and Pediatric Ophthalmology) accounted for 6.2%.

**Fig. 1:**
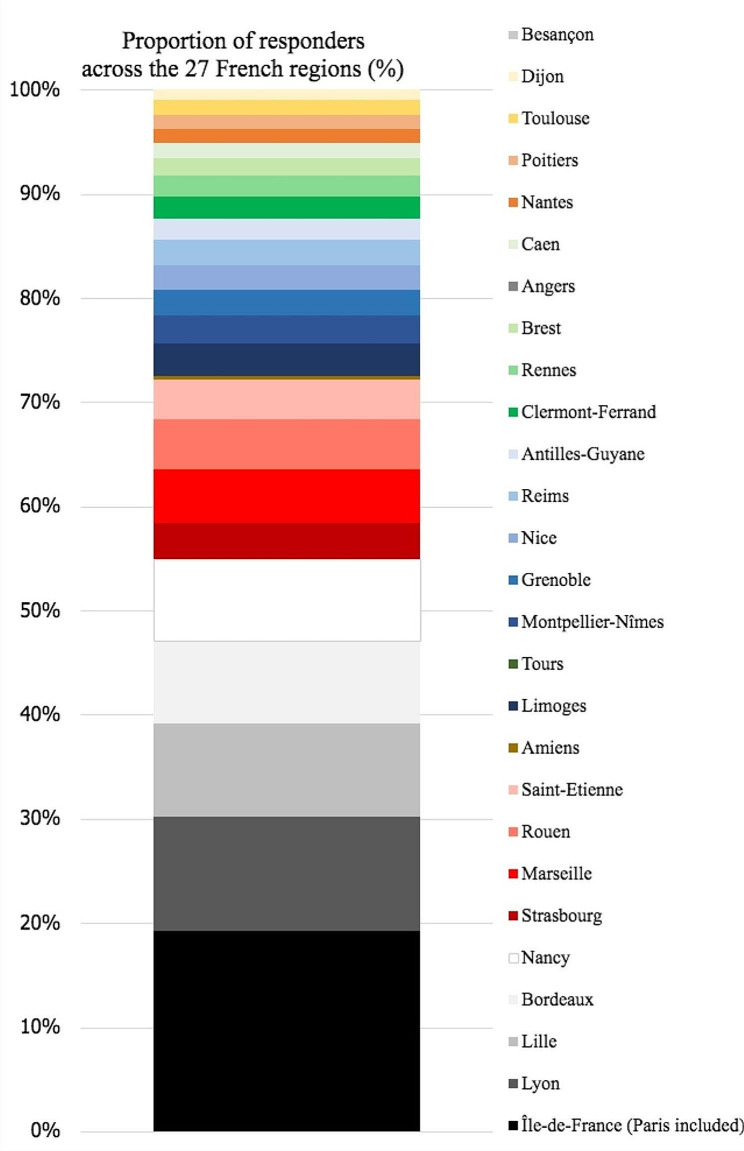
Respondent residents by region of residency

The proportion of respondents distributed homogeneously for seniority (Table 1). Answers converged from the 27 French regions (Fig. 1). A fourth of the answers went from residents of Paris (17%) and Lyon (10%), the two most populated cities in France, followed by Lille (8%) and Bordeaux (7%).

**Table 1:** Seniority of responding resident by year of residency

Seniority within residency program		Individual answers collected		
Phase name	Seniority (year)	Respondents (n=)	Total contacted (n=)	Ratio (%)
*First phase*	*1*	*24*	*154*	*16%*
*Second phase*	*2*	*52*	*152*	*34%*
	*3*	*54*	*151*	*36%*
	*4*	*61*	*152*	*40%*
*Third phase*	*5*	*57*	*155*	*37%*
	*6*	*56*	*141*	*40%*
*Others (Graduates of 2016 or before)*		*17*	*152*	*11%*
*Total*		*321*	*1057*	*30%*

**Fig. 2:**
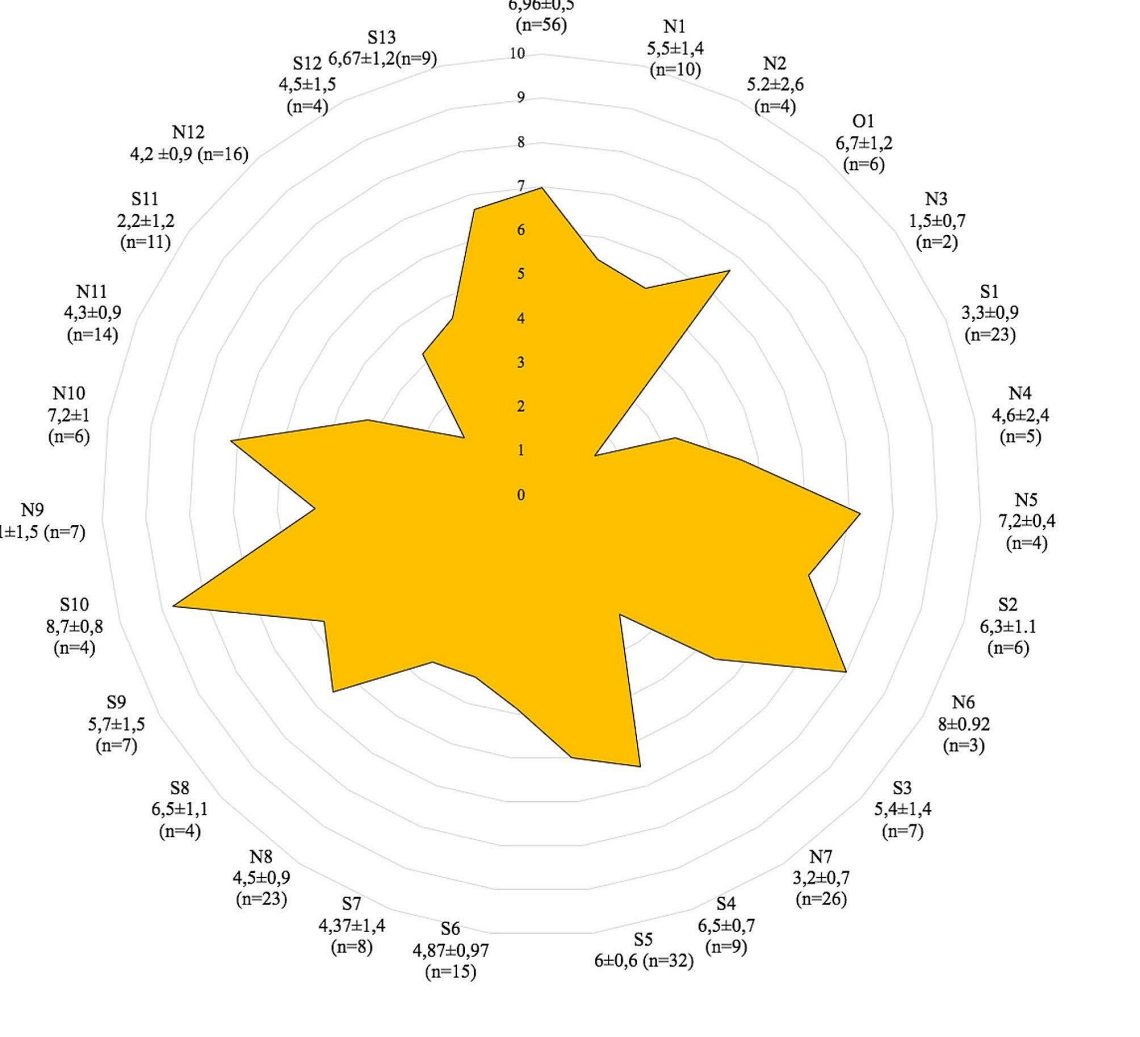
Subjective rating of surgical training during residency according to region from 0 (lowest score) to 10 (greatest score); (mean ± standard deviation)

Respondents attributed a mean score of 5.27 ± 2.4/10 to the surgical training program they were attending to. It included simulations with drylabs wetlabs and hands-on training. The mean score showed great variability depending on the region (Fig. 2).

Regardless of the nature of the rendered ocular surgery, simulation was accessed by all residents in Paris and by 78.1% in other regions (*p* < 0.005). For cataract surgery, the global subjective rate was 7.31 ± 1.89/10 among 186 residents for drylabs and 6.39 ± 2.1/10 among 311 residents for wetlabs training, all simulated surgery taken together.

Answers to binary (yes/no) questions are shown in Table 2. A small majority of respondents (*n* = 193/321; 60.1%) declared to participate to drylabs and/or wetlabs on an optional basis, while more than a quarter (*n* = 113/321; 35.2%) shall attend to simulation program as a formal part of the residency, through skills lab for example. These skills labs were mainly training for cataract surgery and stitching. Only residents from the overseas region of Antilles-Guyane did not have any access to simulation.

**Table 2:** Answers collected for binary questions (ratio of positive answers); FST: “formation spécialisée transversale”. It refers to transversal subspecialty training as an option of residency, basically for Oculoplastics. IdF: Île-de-France (region of Paris and surrounding universities)

Topic investigated	Answers from residents (ratio yes/no)			*p*
	France	IdF	Interrégions	
FST option ?	6,2	5,4	6,4	0.766006
Mentored 1 to 1 ?	48,9	62,5	38,1	0.000792
Identified surgical goals & objectives ?	82,2	76,8	78,5	0.778927
Have access to surgical dry or wet labs?	84,7	100	78,1	0.001194
Have access to basic drylab (kitaro kit)?	23,7	21,4	24,2	0.663244
Access to vitreoretinal surgical simulator?	44,2	76,8	37,4	< 0.00001
Completed cataract surgery as a whole?	80,7	83,9	80	0.498634
Completed part(s) of vitreoretinal surgery?	26,8	44,6	23	0.000049
Would you feel surgically autonomous by the beginning of your 5th year of residency?	57,3	80,4	52,5	0.000125
Would you feel surgically autonomous by the end of your 6th year of residency?	89,4	94,6	88,3	0.161208

A large majority of residents already had performed at least a single step of an ocular surgery on a real patient (Total *n* = 300/321; 93.4%; Paris *n* = 49/56; 87.5%; other regions *n* = 251/265; 94.7%, *p* = 0.047), and *n* = 259/321 (80.7%) out of them claimed having completed a whole cataract extraction procedure (Paris *n* = 47/56; 83.9%, other regions *n* = 212/265; 80%, *p* = 0.498). On average, the first cataract surgery was completed in its entirety by the end of the third semester (Total: 3.8 ± 1.9 semesters; Paris: 2.6 ± 1.4 semesters; regions: 4.05 ± 1.96 semesters, *p* < 0.0001).

Meanwhile, 26.8% (*n* = 86/321) of the respondents had performed at least a procedural step of vitreo-retinal surgery (Paris *n* = 25/56; 44.6%, other regions *n* = 61/265; 23%; *p* = 0.00005), on average at the beginning of the sixth semester (Total: 6.38 ± 1.96 semesters; Paris: 5.2 ± 1.9 semesters; regions: 6.97 ± 1.6 semesters, *p* = 0.0003).

Globally, less than a half of respondents accessed to simulation for vitreoretinal surgery (44.2%). A higher rate of access was claimed by 76.8% of the resident in Paris, compared to 37.4% in other regions (*p* < 0.00001). Less than a ¼ of respondents accessed to training kits (usually KITARO ® kit) during residency, which utility was rated 5.6/10.

Almost a half of the residents (48.9%) were able to identify a senior mentor dedicated to their surgical training, more likely in Paris (Paris *n* = 35/56; 62.5% vs. other regions *n* = 101/265; 38.1%, *p* = 0.00079). Residents accorded themselves in the final comments on the need to benefit from a senior mentoring all along the surgical teaching program. Unsurprisingly, 82.2% of respondents nationwide could not clearly define objectives related to the surgical training program they belong to. In this regard, no significant difference was reported between Paris and other regions (*p* = 0.77).

**Fig. 3:**
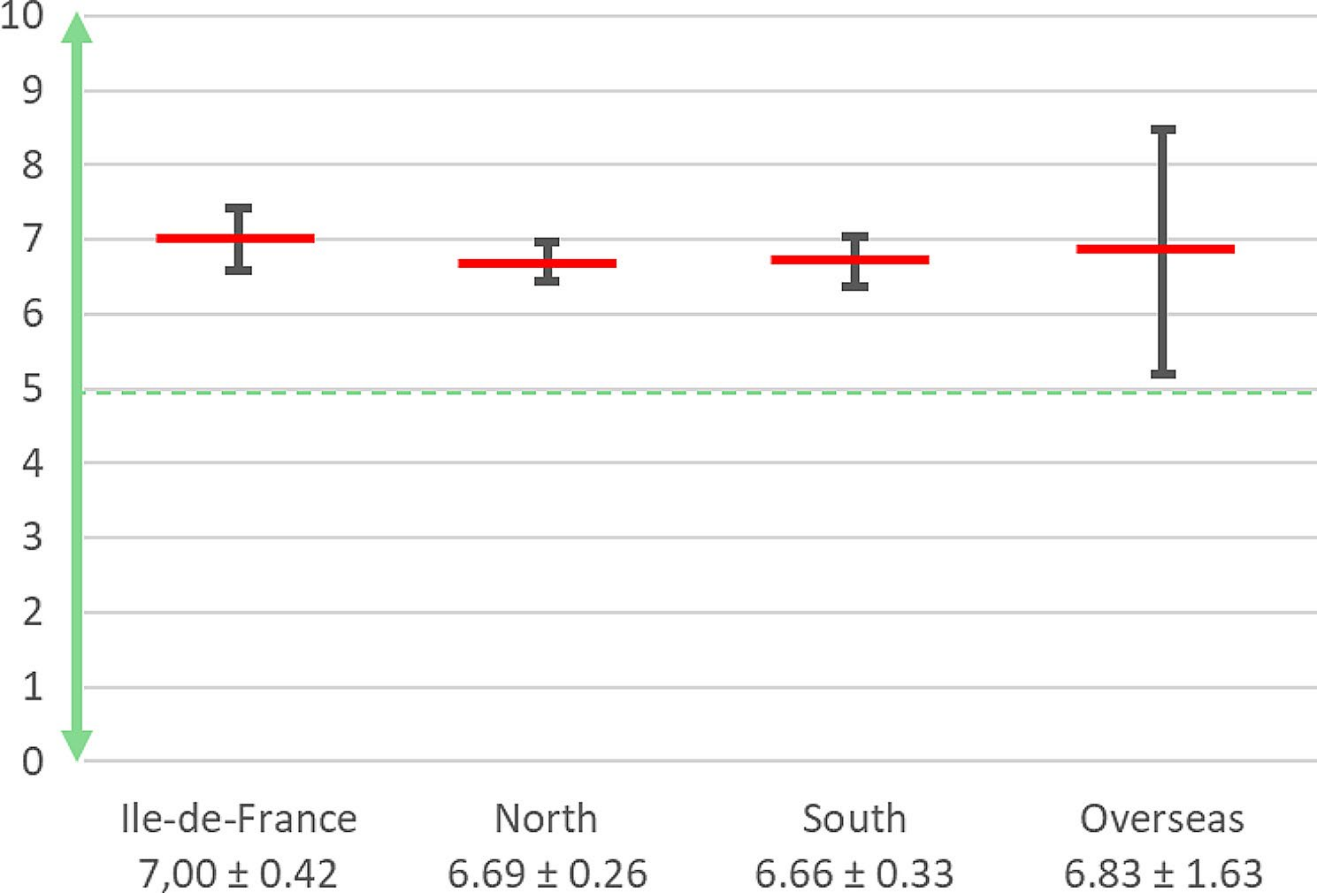
How would residents ideally balance between simulation and hands-on training (0 for simulation exclusively; 10 for hands-on training exclusively)?

Before reaching surgical self-autonomy, only 58 respondents (18%) would balance the surgical training between simulation and hands-on training in a 50:50 proportion, while a quarter considered 30:70 as optimal. Most of the other respondents (*n* = 233; 72.58%) suggested favoring hands-on over simulation along residency (Fig. 3). At the same time, hands-on surgical training during residency was given an overall score of 5.5 ± 2.6/10. The surgical hands-on training rate suffered from great disparities between Paris and other regions (Paris: 7.1 ± 2.2/10, Regions: 5.2 ± 2.5/10, *p* < 0.0001).

Residents foresaw being surgically independent significantly more by the end of the third phase than by the end of the second phase (*n* = 287/321; 89.4% vs. *n* = 184/321; 57.3%, *p* < 0.00001). We noticed a discrepancy between Paris and other regions at the end of the 8th semester only (by the end of second phase semester: Paris *n* = 45/56; 80.4%; other regions *n* = 139/265; 52.5%; *p* = 0.000125; 10th semester: Paris *n* = 53/56; 94.6%; other regions *n* = 234/265; 88.3%; *p* = 0.16). Some of the residents mentioned an easier access to hands-on surgery in private structures, mostly residents outside the Paris (Île-de-France) region. Others reported that suburban hospitals allowed easier access to surgery compared to their related university center. They commented on the lack of national standardization, as well as the need for more formal surgical aims and senior mentoring. They would suggest a mentoring at least by the 3rd month of residency. Naturally, they would welcome a better access to surgery on real patients. They expressed some regrets about the lack of evaluation of teaching programs and suggested that the quality of surgical pedagogy should be evaluated by residents through a formal rating. They valued more videotaped surgical courses provided by expert surgeons. They would further welcome a pedagogical debriefing based on their own videotaped surgical performances. The need for more theory in a top-down style was not approached.

In the present survey, residents seemed to favor experiential practice based on simulation, followed by hands-on training. Access to simulation followed by a transition towards hands-on surgical training was of strong demand from residents who didn’t access to it.

Other comments also complained about the too selective access during surgical training to subspecialty, such as corneal, glaucoma, or vitreoretinal surgery.

## Discussion

The satisfaction of residents in ophthalmology towards surgical training is encouraging and globally positive throughout France. Residents valued simulation training. It seems in line with what has been reported from the specific area of Paris-Île-de-France by Martin et al. [9]

With a third of responders among the total of contacted residents, the collected declarative data can be interpreted as representative in the present study. It also corroborates that all French ophthalmology residency programs were participating in the study.

At a national level, we observed significant disparities between Paris and other regions as well as between regions themselves. It reflects a poor level of standardization nationwide for surgical training programs. According to the provided answers, the access itself to surgical training suffers from high variability in quality and for quantitative availability.

We did not focus on the causes of such disparities. The lack of standardization at the national level has been reported by other international studies [8]. It is likely that the lack of a clear national standardization for surgical training, for example through surgical goals & skills textbooks, might play a role. Rating the objectives of surgical learning programs has been proposed using the method of SMART (specific, measurable, achievable, reasonable, and time-bound) goal framework [10]. Recent studies have evaluated residency programs at the European level [11]. They determined the minimal number of procedures to be performed for each type of surgery during residency. Surgical volume is an important metrics to approach the residents’ ease to perform a specific procedure, but the ability to operate should also be determined by a senior surgeon. Learning quality of proceeding and other non-technical skills is also pivotal.

Residents could take advantage of a textbook of goals & objectives. It would serve as a tool to design surgical supervision. It would also contribute to prove surgical achievements, thus putting senior surgeons more at ease to let residents operate. Such a tool has already been discussed in the literature, [12] and can further complete a surgeon’s certification (operating license). At the same time, it would credit residents for more access to the operating room by endorsing the role of leading operator for instance.

In the United States, the ACGME (Accreditation Council for Graduate Medical Education) cleared guidelines for teaching during residency. It is in charge to enforce compliance to guidelines for residency programs. Residents are also interrogated annually, through a questionnaire, which figures their satisfaction with the residency program [13]. However, whether such feedback could be meaningful and even elaborated remains to be determined in countries, either poorly allocated for teaching programs or less centrally coordinated.

Responding residents globally described a poor access to subspecialty surgical training, in accordance with previous data [6, 7, 14]. The opportunity to practice as a subspecialist is limited compared to comprehensive ophthalmology. The more specialized the practice is, the tougher is teaching complexity. Besides, less patients are referred to subspecialists. Logically, subspecialized practice is sparsely accessed during fellowships, even potentially at a senior level. Although residents are complaining about it when interviewed, less surgeons are needed in the field. As a matter of fact, only a few future surgeons should be specifically trained. It seems then acceptable that career history of excellence should rule access to it.

Our questionnaire did not include questions regarding surgery in emergency situations, such as identifying dystopic anatomy and suturing recent open globe injuries, but we postulate that the same approach could apply for such complex procedures.

Surgical teaching has progressively evolved from relying on the Halstedian model of graduate responsibility to surgery simulation as a preliminary step in the learning course [15]. Higher standard for patient safety added to less teaching resources may have prompted the transition [16]. Simulation now serves as a key element for transition towards hands-on surgical training. The benefit has been widely demonstrated in the past decade, either using the *EyeSI simulator* [3, 17–21] or throughout other wetlabs [22, 23]. However, our study enlightens regional disparities to access simulation (drylabs and wetlabs). In French regions, accessibility varies greatly, depending on the involvement of local universities and health agencies (ARS, Agences Régionales de Santé). As a matter of fact, not all regions have a simulation platform available. In the meantime, Paris region set dry- and wetlabs widely available to residents through virtual reality surgical simulators and in-training workshops, placing simulation as a mandatory part of the resident’s preclinical training.

Obviously, we acknowledge several limitations in the present study. It is retrospectively designed. As a questionnaire optionally taken, all French residents could not be exhaustively interviewed. Nevertheless, we are grateful that a third of the residents took our questionnaire, which is meaningful for an opinion-based study. Answers were subjective. They may also reflect the lack of knowledge of residents on their access to simulation or surgery, especially among younger residents.

It is possible that some respondents to the questionnaire sent answers twice, although this eventuality seems very unlikely, given the time consumed to fill such a questionnaire, among residents, who are dealing with busy schedules in clinical practice. We would have also detected identical charts in our database in such an occurrence.

In conclusion, French Ophthalmology residents claimed satisfaction with the surgical training program they belong to, along with some regional disparities. The need for harmonization of surgical goals and objectives is underlined. The access to simulation was valued by residents, based on a progressive and supervised transition to surgical training on real patients.

Residents would support the evaluation of surgical skills, which could serve residents as an “operating license”, attest of the specific surgical knowledge they acquired and prompt their access to real surgery mastered by seniors. According to residents in ophthalmology, the program they are enrolled in should be evaluated by themselves, to improve surgical teaching.

### Electronic supplementary material

Below is the link to the electronic supplementary material.

Supplementary Material 1

## Data Availability

The datasets used and/or analysed during the current study are available from the corresponding author on reasonable request.

## Abbreviations

ARS: Agence Régionale de Santé (French regional health agency)
FST: “formation spécialisée transversale”. It refers to transversal subspecialty training as an option of residency, basically for Oculoplastics
IdF: Île-de-France (region of Paris and surrounding universities)
