# Supplementary material for: Preclinical training of future ocular surgeons: a French opinion-based study
Source: BMC Med Educ. 2024 Feb 9;24:129. doi: 10.1186/s12909-024-05124-8 (PMC10858601; doi:10.1186/s12909-024-05124-8)
Supplement: Supplementary file 1 — Supplementary Material 1 [file 12909_2024_5124_MOESM1_ESM.docx]

# Supplementary material:

# Preclinical Training of Future Ocular Surgeons: Questionnaire content

1 – What is the region of your residency program?

2 – When was the year of your enrollment in the residency program of ophthalmology?

3 – Are you enrolled (or did you ever participate) in a FST program (Formation spécialisée tranvsersale)? (Yes/No)

4 – How would you rate the surgical training of your residency program, taking in account both simulations and surgery on patients in real life, from 0 (insufficient) to 10 (very satisfactory)?

5 – Before reaching full autonomy for basic ocular surgery, how would you ideally balance your surgical training between simulation and hands-on training: 0 =more simulation to 10=more hands-on training?

6 – About surgery on patients in real life: can you identify one (or a few) mentor(s) who are directly in charge of your supervision for surgical training in the operating room? (Yes/No)

7 – About surgery on patients in real life: Are you assigned to a list of surgical goals & objectives to reach by the end of the residency program? (Yes/No)

8 – Surgical simulation with drylabs and wetlabs: In your residency program, does training with simulation participate in your ability to operate on patients as a compulsory part (like an“operating license”)/ as an optional training/is not available/other?

9 - Surgical simulation with drylabs and wetlabs: For your surgical training, is simulation [permanently / occasionally] offered by [the residency program itself/private companies] and are simulation labs free of charge for you [yes/no]?

10 - Surgical simulation with drylabs and wetlabs: how would you rate simulation labs’ availability during residency, from 0 (not available) to 10 (permanently available)?

11 – What kind of simulators are available on your training platform: synthetic eyeball/biologic eyeball/synthetic part of an eyeball/Virtual reality eyeball/ no access to simulation at all?

12 – What kind of procedure could be simulated on your training platform: steps of cataract surgery /complete cataract surgery procedure/suturing/filtering surgery/vitreoretinal surgery?

13 – If you have access to it, how would you rate the pedagogic value of the wetlabs, from 0 (poor) to 10 (the highest possible)?

14 – Do you have access to any Virtual Reality (VR) surgical simulator for drylab? (Yes/No)

15 – If you have access to it, how would you rate the pedagogic value of VR drylabs, from 0 (poor) to 10 (the highest possible)?

16 – Do you have access to Kitaro kits (FCI Ophthalmics; MA, USA) during residency?

17 – If you have access to it, how would you rate the pedagogic value of Kitaro kits from 0 (poor) to 10 (the highest possible)?

18 – Do you have access to a vitreoretinal surgery simulator?

19 – If you have access to it, how would you rate the pedagogic value of the vitreoretinal surgery simulator, from 0 (poor) to 10 (the highest possible)?

20 – When did you perform your first ocular surgery on a patient in real life (any type, steps or whole surgery)?

21 – When did you completed your first cataract surgery in real life, as a whole?

22 – If your first cataract surgery was performed as a whole during residency, when did it happened (seniority within the residency program)?

23 – When did you perform your first vitreoretinal surgery (one step at least) on a patient in real life (not yet/during residency)?

24 – If you performed your first vitreoretinal surgery (at least one step) on a patient during residency, when did it happened (seniority within the residency program)?

25 – Do you expect autonomy for cataract surgery by the beginning of your fifth year of residency (beginning of “consolidation phase”)?

26 – Do you expect autonomy for cataract surgery by the end of your sixth year of residency (end of “consolidation phase”)?

27 – Can you comment on your surgical training in real life on patients during residency (“consolidation phase” included)?

28 – Can you comment on your surgical training during residency, or to optimize the training for future ocular surgeons?
